# Integrated Analysis of lncRNA-Mediated ceRNA Network Reveals a Prognostic Signature for Hepatocellular Carcinoma

**DOI:** 10.3389/fgene.2020.602542

**Published:** 2020-12-14

**Authors:** Jian-Rong Sun, Chen-Fan Kong, Kun-Min Xiao, Jia-Lu Yang, Xiang-Ke Qu, Jing-Hui Sun

**Affiliations:** ^1^Department of Clinical Medicine, Beijing University of Chinese Medicine, Beijing, China; ^2^Oncology Department of Integrated Traditional Chinese and Western Medicine, China-Japan Friendship Hospital, Beijing, China; ^3^Gastroenterology Department, Dongzhimen Hospital, Beijing University of Chinese Medicine, Beijing, China

**Keywords:** hepatocellular carcinoma, long non-coding RNA, competing endogenous RNA, prognostic signature, nomogram

## Abstract

Hepatocellular carcinoma (HCC) is one of the most common types of malignancy and is associated with high mortality. Prior research suggests that long non-coding RNAs (lncRNAs) play a crucial role in the development of HCC. Therefore, it is necessary to identify lncRNA-associated therapeutic biomarkers to improve the accuracy of HCC prognosis. Transcriptomic data of HCC obtained from The Cancer Genome Atlas (TCGA) database were used in the present study. Differentially expressed RNAs (DERNAs), including 74 lncRNAs, 16 miRNAs, and 35 mRNAs, were identified using bioinformatics analysis. The DERNAs were subsequently used to reconstruct a competing endogenous RNA (ceRNA) network. A lncRNA signature was revealed using Cox regression analysis, including LINC00200, MIR137HG, LINC00462, AP002478.1, and HTR2A-AS1. Kaplan-Meier plot demonstrated that the lncRNA signature is highly accurate in discriminating high- and low-risk patients (*P* < 0.05). The area under curve (AUC) value exceeded 0.7 in both training and validation cohort, suggesting a high prognostic potential of the signature. Furthermore, multivariate Cox regression analysis indicated that both the TNM stage and the lncRNA signature could serve as independent prognostic factors for HCC (*P* < 0.05). Then, a nomogram comprising the TNM stage and the lncRNA signature was determined to raise the accuracy in predicting the survival of HCC patients. In the present study, we have introduced a ceRNA network that could contribute to provide a new insight into the identification of potential regulation mechanisms for the development of HCC. The five-lncRNA signature could serve as a reliable biosignature for HCC prognosis, while the nomogram possesses strong potential in clinical applications.

## Introduction

Liver cancer is one of the most common types of malignancy, with 841,080 new cases and 781,631 cancer deaths being reported globally in 2018 (Bray et al., 2018). In the United States, the death rates of liver are the fastest growing ones in comparison to other cancer (Islami et al., 2017). It is estimated that 42,030 new cases of liver cancer will be diagnosed in 2019 causing 31780 deaths in United States (Siegel et al., 2019). The major histology phenotype (approximately 80%) of liver cancer is hepatocellular carcinoma (HCC) accounting for more than 80% of liver cancer cases worldwide (McGlynn et al., 2015; Yang et al., 2019). HCC usually occurs in people with chronic liver injury, primarily as a result of a combination of chronic infection of hepatitis B virus (HBV), hepatitis C virus (HCV), alcohol abuse, aflatoxin, smoking, or metabolic syndrome. Traditional therapies for HCC, such as surgery, radiation therapy, chemotherapy, and radiofrequency ablation therapy, are not as effective as expected. Although considerable progress has been made in the diagnosis and therapy of HCC in the past 10 years, the prognosis is still not satisfactory. Therefore, it is urgently important to identify novel biomarkers that can more accurately prognose the outcomes of the disease to optimize the clinical management of HCC patients (Bruix et al., 2016).

Long non-coding RNAs (lncRNAs) are transcripts with little or no protein-coding potential (Chi et al., 2019). The lncRNAs are larger than 200 nucleotides in length and can function similarly to RNAs or proteins due to their secondary and three-dimensional structures (Novikova et al., 2013; Qian et al., 2019). Recent studies have demonstrated that lncRNAs can play a vital role in cancer through multiple molecular mechanisms (Deans and Maggert, 2015; Deng et al., 2017; Tang et al., 2017; Wu et al., 2017; Xu et al., 2018). For instance, the overexpression of SNHG6 was associated with poor prognosis and can enhance migration, invasion, and metastasis in tumors (Wang et al., 2020). A high expression level of the lncRNA URHC can facilitate proliferation and inhibit apoptosis in HCC by activating the ERK/MAPK pathway (Xu et al., 2014). Moreover, the competing endogenous RNA hypothesis suggested that lncRNAs could function as endogenous molecular sponges that competitively bind miRNAs via shared microRNA response elements (MREs) with reverse complementary binding seed regions to indirectly regulate the expression levels of downstream mRNAs and subsequently participate in cancer development (Yang et al., 2016; Bai et al., 2019). For instance, the lncRNA HOTAIR could increase the expression of HER2 by competing with miR-331-3p, thereby promoting cancer progression and metastasis (Liu et al., 2014). LncRNA SNHG6-003 was associated with shorter survival in HCC demonstrating its ability to facilitate cell proliferation by binding with miR-26a/b/TAK1 (Cao et al., 2017). The relationship between lncRNAs and cancer progression has been proven from various recent research works. Moreover, the development of genome-wide sequencing technologies has accelerated the identification of prognostic markers for HCC (Lin et al., 2018; Zhang et al., 2020; Zhao et al., 2020). However, a nomogram combining the molecular biomarkers with clinical characteristics has not been determined yet.

HCC-associated gene expression profiles were retrieved from the TCGA database in the present study. The DERNAs, including those encoding mRNAs, lncRNAs, and miRNAs, were identified using bioinformatic methods. A lncRNA-miRNA-mRNA regulatory network was introduced following the ceRNA hypothesis. Next, functional and pathway enrichment analysis of the mRNAs involved in this regulatory network was conducted. Then, a 5-lncRNA prognostic signature was verified and validated using the ceRNA network. Finally the independent prognostic factors were analyzed and combined to a nomogram that was proven to be highly accurate in predicting the survival rate of HCC patients.

## Materials and Methods

**FIGURE 1:**
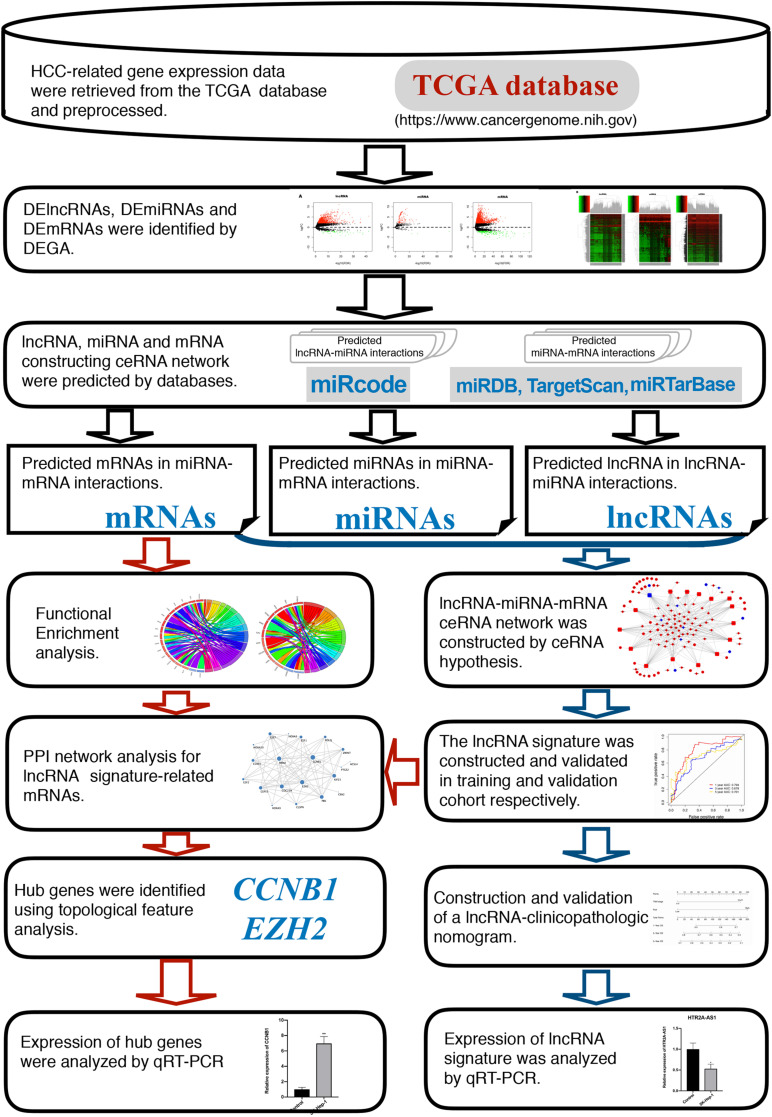
Flow chart of the current study.

The flowchart of this study is presented in Figure 1.

### Data Acquisition

The RNA sequencing (RNA-seq) data (FPKM, Fragment Per Kilobase of transcript per Million mapped reads) and the corresponding clinical information (including age, gender, tumor grade, TNM stage) of 376 HCC patients were downloaded from the TCGA database^1^. In particular, this study obtained a total of 426 samples, containing 376 HCC samples and 50 adjacent normal samples. This study was conducted exclusively following TCGA guidelines. Considering that our data were derived from the TCGA, consent from the ethics committee was not necessary.

### Data Processing and Differentially Expressed Gene Analysis (DEGA)

The RNA-seq data of HCC were processed and normalized with a trimmed mean of *M*-values (TMM). R “edgeR” (version 3.6.0), a bioconductor package for differential expression analysis of digital gene expression data, was used to identify differentially expressed (DE) lncRNAs, miRNAs, and mRNAs in the comparison between the adjacent normal and HCC samples, respectively (Robinson et al., 2010). The RNAs with FDR less than 0.05 and | logFC| exceeding 2 were considered to be DERNAs. Heatmap and volcano plots were used to visualize the gene expression profiles of the different RNAs that were measured and the results of the differential expression analysis between normal and HCC samples, respectively.

### Construction of the ceRNA Interaction Network

A lncRNA–miRNA–mRNA ceRNA interaction network has been reconstructed following the ceRNA hypothesis. The DElncRNA targets of miRNAs were mined from the miRcode database (version 11, June 2012) (Jeggari et al., 2012), to obtain the lncRNA-miRNA interactions and use them to construct the ceRNA network. Data from three databases, including miRDB (version 6.0, June 2019), miRTarBase (version 7.0, September 2017), and TargetScan (version 3.1, October 2006), were integrated to predict the corresponding mRNA targets of miRNAs (Fromm et al., 2015; Wong and Wang, 2015; Chou et al., 2018). Subsequently, the predicted miRNA:mRNAs targets were combined with the DEmRNAs to obtain the final miRNA-mRNA interactions. Only the miRNA-mRNA interactions found in all three databases were selected as candidate genes for the construction of the ceRNA network to increase the reliability of the results. Finally, Cytoscape software (Version 3.7.1) was employed to visualize the lncRNA–miRNA–mRNA ceRNA network.

### Function Enrichment Analysis

The Database for Annotation, Visualization and Integrated Discovery (DAVID) online functional annotation tool^2^ was used to perform the Gene Ontology (GO) and Kyoto Genome Encyclopedia (KEGG) pathway enrichment analysis on the mRNAs of the reconstructed network. The cut-off criterion of significant GO terms and KEGG pathways was a *p*-value threshold of 0.05. The R “ggplot2” (version 3.2.1) and the “GOplot” (version 1.0.2) packages were then used to visualize the results of functional and pathway enrichment analysis.

### Construction and Validation of the Prognostic lncRNA Signature Based on the ceRNA Network

The 376 HCC patients were randomly divided into training and validation datasets by the R “caret” package (version 6.0-86), an R software package usually used for data training and grouping in regression analysis. Univariate Cox regression analysis was performed in the training cohort to screen the lncRNAs that were associated with the overall survival (OS) of HCC patients. Subsequently, the lncRNAs exhibiting statistically significant differences (*P* < 0.01) in univariable Cox regression analysis were selected for multivariate Cox regression analysis to identify the independent prognostic lncRNAs and build the lncRNA signature. The risk score formula of the lncRNA signature was constructed as follows:

RiskScore=βel⁢n⁢c⁢R⁢N⁢A1*xp+l⁢n⁢c⁢R⁢N⁢A1βel⁢n⁢c⁢R⁢N⁢A2*xp+l⁢n⁢c⁢R⁢N⁢A2⋯βel⁢n⁢c⁢R⁢N⁢A⁢n*xpl⁢n⁢c⁢R⁢N⁢A⁢n

where N is the number of prognostic lncRNAs, exp is the expression value of a lncRNA, and β is the coefficient of a lncRNA in the multivariate Cox regression model. Every patient in the training cohort was assigned with a risk score using the aforementioned formula and then classified to one of two subgroups (high- and low-risk) according to the median risk score. Kaplan-Meier analysis and the log-rank test were conducted using the R “survival” package, an R software package used for survival analysis including Kaplan-Meier curves and Cox models, to study the ability of the biosignatures to discriminate the high- and low-risk groups. The predictive ability of the lncRNA signature was evaluated by computing the area under the curve (AUC) value at 1, 3, and 5 years survival of the receiver operating characteristic (ROC) curve using the survival ROC package (version 1.0.3), an R software package used for plotting ROC curve of survival data. The prognostic lncRNA signature was then validated in the validation and additional external cohorts, respectively.

Finally, stratified Cox survival analysis was performed to verify the prognostic power of the final signature in such cohorts in comparison to age, gender, TNM stage, and tumor grade.

### Development and Validation of the lncRNA-Clinicopathological Nomogram

A nomogram is a statistical predictive tool that combines multiple prognostic factors to assess the survival probability in individual patients (Iasonos et al., 2008). Thus, univariate Cox regression analysis and multivariate Cox regression analysis were performed to investigate whether the lncRNA signature along with all clinical variables described above were independent prognostic factors of HCC. Finally, the variables with a *p* < 0.05 in multivariate Cox regression analysis were selected to develop a nomogram model that can improve the accuracy of the prediction of the survival probability of HCC patients. The C-index and calibration plot were used to evaluate the discrimination and calibration abilities of the nomogram. A calibration curve close to 45° is an indication of the good predictive ability of the model constructed by this factor. Finally, the TNM model, lncRNA signature and the nomogram model combining the TNM stage with the lncRNA signature were compared by ROC curve, dynamic AUC value, C-index and decision curve analysis (DCA) to explore whether the nomogram possesses better predictive power and clinical applications than other models.

### Protein-Protein Interaction Network Construction and Survival Analysis

The mRNAs in the ceRNA network, which were associated with lncRNAs to construct prognostic signatures, were selected to conduct protein-protein interaction (PPI) network analysis using the STRING Database and web-tool^3^. The minimum required interaction score was set as 0.9 to obtain more reliable results. The screened interaction pairs were utilized to re-construct the PPI network and this network was visualized with Cytoscape software (Version 3.7.1), a Network graphics drawing software. Topological feature analysis was then performed to identify hub genes by the CentiScaPe2.2 plugin of Cytoscape software. CentiScaPe2.2 is a plugin used for network analysis based on topological and biological properties. Furthermore, Kaplan-Meier survival analysis and the log-rank test were employed to explore the association between hub genes and HCC prognosis.

### Validation of the lncRNA Signature and Hub Genes by Relative Quantitative Real-Time PCR (qRT-PCR)

The expression levels of the lncRNA signature and two hub genes were measured in a control cell line (hiPS-HEP) and four HCC cell lines (SK-Hep-1, HepG2, Hep3B, HuH-7) to validate the reliability of our results. The human-induced pluripotent stem (iPS) cell-derived hepatocyte (hiPS-HEP) kit was purchased from Takara Bio (Cat. No. Y10133) and then induced and cultured according to the user manual. Human hepatocellular carcinoma (HCC) cell lines (SK-Hep-1, HepG2, Hep3B, HuH-7) were obtained from the National Infrastructure of Cell Line Resources (Beijing, China). HCC cells were cultured in Dulbecco’s modified Eagle’s medium (DMEM, Gibco, United States), 10% fetal bovine serum (FBS, Gibco, United States), and 1% penicillin/streptomycin (Gibco, Canada). All HCC cells and hiPS-HEP cells were cultured at 37°C with 5% CO_2_. Total RNA was extracted from cells using the RNeasy Mini Kit (Qiagen, United States. Cat. 74104), and reverse transcription was subsequently performed using the 5^∗^ All-in-one RT MasterMix (ABM, US. Cat. No. G492). qRT-PCR was performed with a SYBR Green Real-time PCR Kit (Keygen Biotech, Nanjing, China, Cat. KGA1339-1) on a QuantStudio 5 Real-Time PCR System (Thermo Fisher Scientific, United States). All experiments were repeated at least three times. The RNA primer sequences are listed in Supplementary Table 1. Relative expression was calculated using the comparative threshold cycle (Ct) method.

## Results

### Identification of Differentially Expressed lncRNAs, miRNAs, and mRNAs

The clinical data of HCC patients was shown in Supplementary Table 2. A total of 1044 DElncRNAs (986 up- and 58 downregulated), 126 DEmiRNAs (123 up- and 3 downregulated), and 1981 DEmRNAs (1774 up- and 207 downregulated) were identified conducting differential expression analysis between HCC and non-tumor samples and using FDR of 0.05 and absolute Log Fold Change of 2 as thresholds to infer statistically significant changes (Supplementary Tables 3–5). The differential expression analysis results of the DElncRNAs, DEmiRNAs and DEmRNAs were visualized using volcano plots and heatmaps (Supplementary Figure S1).

### Establishment of the ceRNA Network

To elucidate the potential regulatory mechanism governing ceRNA in HCC, we established a ceRNA network for HCC based on the ceRNA hypothesis. After exploiting miRcode, a total of 279 lncRNA-miRNA interaction pairs were identified, including 74 lncRNAs and 16 miRNAs (Supplementary Table 6). Next, we predicted the potential miRNA-mRNA interaction pairs by 3 databases containing miRDB, miRTarBase, and TargetScan. As a result, 41 miRNA-mRNA pairs were detected (Supplementary Table 7). Finally, according to the interaction pairs of lncRNA-miRNA and miRNA-mRNA, we constructed a lncRNA-miRNA-mRNA ceRNA network consisting of 74 lncRNAs, 16 miRNAs, and 35 mRNAs involved in this network. Moreover, the ceRNA network was visualized by Cytoscape (Supplementary Figure S2).

### GO and KEGG Enrichment Analysis

**FIGURE 2:**
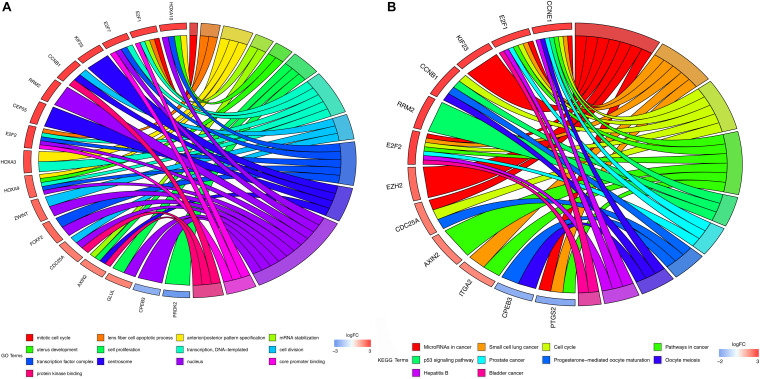
GO and KEGG enrichment analysis. **(A)** GO enrichment analysis of mRNAs in ceRNA network. **(B)** KEGG pathway analysis of mRNAs in ceRNA network. In each chord diagram, the enriched genes are shown on the left and the enriched GO or KEGG clusters are shown on the right. The down-regulated mRNAs are shown in blue whereas the up-regulated mRNAs are shown in red. Each item is represented by a colored bar.

The DAVID tool was applied to perform GO enrichment analysis and KEGG pathway enrichment to promote the understanding of the biological function of mRNAs included in the ceRNA network. 13 significant GO functional terms (*P* < 0.01) were identified demonstrating that HCC is primarily enriched in the mitotic cell cycle and cell cycle (Figure 2A). The KEGG enrichment analysis identified 10 significant KEGG pathways (*P* < 0.05) with most of them being associated with oncogenesis (Figure 2B).

### Construction and Validation of the lncRNA Signature

**FIGURE 3:**
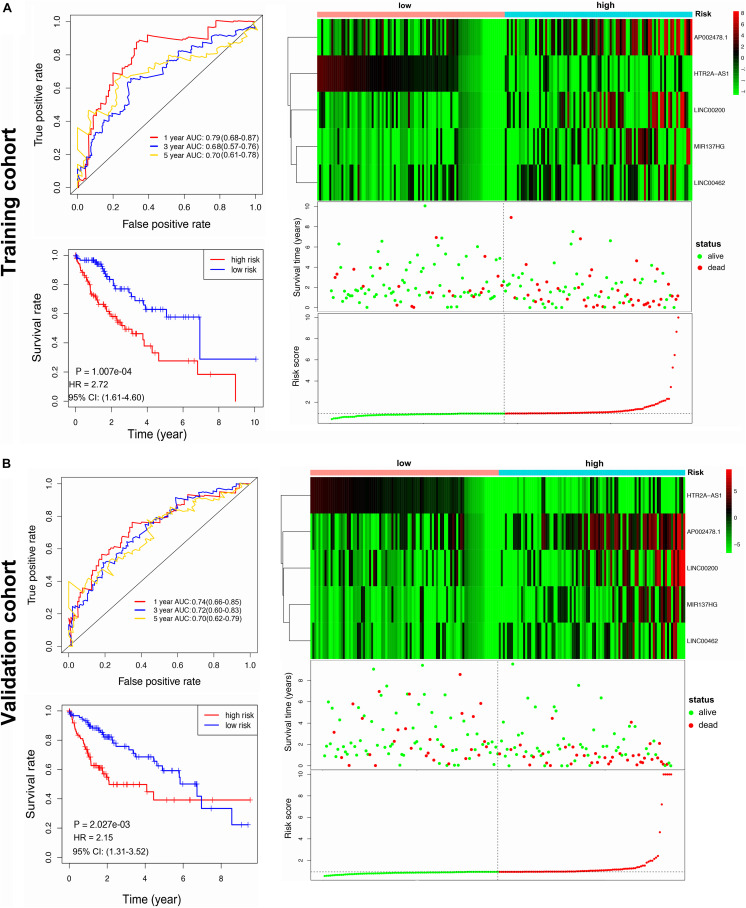
Construction and validation of the lncRNA prognostic signature. **(A)** ROC curve analysis for 1, 3, 5 year OS of HCC, Kaplan-Meier curve of the lncRNA signature, a heatmap of lncRNA signature expression, and risk score in training cohort. **(B)** ROC curve analysis for 1, 3, 5 year OS of HCC, Kaplan-Meier curve of the lncRNA signature, a heatmap of lncRNA signature expression, and risk score in validation cohort.

The HCC patients (*n* = 376) were randomly split into training or validation cohorts, and no significant difference was observed in the clinical characteristics between the two cohorts (Table 1). One hundred eighty-eight patients were included in the training cohort to develop the prognostic signature and train the prognostic models. Univariate Cox regression analysis screened 12 survival-associated lncRNAs (Supplementary Table 8), and these lncRNAs were screened for multivariate Cox regression analysis. As a result, five lncRNAs (i.e., LINC00200, MIR137HG, LINC00462, HTR2A-AS1, and AP002478.1) were selected to construct the prognostic signature. The risk score formula was the following: Risk score = (0.007 × expression of LINC00200) + (0.023 × expression of MIR137HG) + (0.008 × expression of LINC00462) + (−0.071 × expression of HTR2A-AS1) + (0.008 × expression of AP002478.1). Each patient was assigned with a risk score using the lncRNA signature and was then allocated to the high-risk group (*n* = 94) or the low-risk group (*n* = 94) using the median risk score as the classification threshold (Supplementary Table 9). The 1-, 3-, and 5 year OS baseline information were shown in Supplementary Table 10. The AUC values of the ROC curve for 1-, 3-, and 5 year overall survival (OS) were 0.794, 0.678, and 0.701, respectively. The high-risk group presented poor prognosis compared to the low-risk group (Figure 3A).

**TABLE 1:** Clinical characteristics of HCC patients in the training and validation cohort.

	Cohort, No. (%)			
Variables	Training (*n* = 188)	Validation (*n* = 188)	χ^2^-value	*P*-value
**Age (years)**				
< 60	96 (51.1)	87 (46.3)	0.68	0.41
≥ 60	92 (48.9)	101 (53.7)		
**Gender**				
male	122 (64.9)	127 (67.6)	0.19	0.66
female	66 (35.1)	61 (32.4)		
**Tumor grade**				
G1/2	118 (62.8)	124 (66.0)	0.29	0.59
G3/4	70 (37.2)	64 (34.0)		
**TNM stage**				
Stage I/II	127 (67.6)	138 (73.4)	1.28	0.26
Stage III/IV	61 (32.4)	50 (26.6)		
**T stage**				
T1/2	136 (72.3)	140 (74.5)	0.08	0.77
T3/4	52 (27.7)	48 (25.5)		
**N stage**				
N0	125 (66.5)	130 (69.1)	0.32	0.57
N1	63 (33.5)	58 (30.9)		
**M stage**				
M0	130 (69.1)	136 (72.3)	0.12	0.73
M1	58 (30.9)	52 (27.7)		

The performance lncRNA signature was further evaluated in the validation cohort. One hundred eighty-eight patients were assigned to a high-risk group (*n* = 94) and a low-risk group (*n* = 94) by a median risk score threshold (Supplementary Table 9). The 1-, 3-, and 5 year OS baseline information were shown in Supplementary Table 10. The AUC values of the ROC curve for 1-, 3-, and 5 year overall survival (OS) were 0.737, 0.719, and 0.704, respectively. The high-risk group presented a significantly poorer prognosis compared to the low-risk group (Figure 3B).

Moreover, all HCC patients were classified into different cohorts by clinicopathological features, such as age, gender, tumor grade, and TNM stage. Cox survival analysis was then conducted in these stratified cohorts to study the prognostic potential of the lncRNA signature. The results clearly demonstrated that the high-risk group had a worse prognosis than the low-risk group in nearly all cohorts (Table 2). In summary, these results suggested that the lncRNA signature can retain its efficiency for predicting prognosis after corrections for clinical characteristics.

**TABLE 2:** Cox survival analysis of the lncRNA signature in stratified HCC cohort.

Variables	Training cohort		Validating cohort		Combination	
	HR (95% CI)	*P*-value	HR (95% CI)	*P*-value	HR (95% CI)	*P*-value
**Age (years)**						
< 60	3.28 (1.52–7.09)	0.003	2.04 (0.94–4.44)	0.073	2.62 (1.52–4.51)	<0.001
≥ 60	2.20 (1.06–4.56)	0.034	2.16 (1.13–4.11)	0.019	2.14 (1.33–3.44)	0.002
**Gender**						
male	4.45 (2.25–8.8)	< 0.001	2.63 (1.36–5.08)	0.004	3.45 (2.16–5.53)	< 0.001
female	1.09 (0.47–2.56)	0.84	1.53 (0.71–3.28)	0.274	1.27 (0.72–2.21)	0.409
**Tumor grade**						
G1/2	3.03 (1.59–5.78)	< 0.001	1.97 (1.05–3.72)	0.036	2.43 (1.57–3.78)	< 0.001
G3/4	2.33 (0.92–5.88)	0.074	2.70 (1.01–7.23)	0.048	2.51 (1.28–4.91)	0.007
**TNM stage**						
Stage I/II	2.41 (1.19–4.90)	0.015	1.61 (0.85–3.06)	0.147	1.99 (1.25–3.18)	0.004
Stage III/IV	3.69 (1.62–8.43)	0.002	3.83 (1.51–9.74)	0.005	3.44 (1.90–6.22)	< 0.001
**T stage**						
T1/2	2.21 (1.14–4.31)	0.019	1.66 (0.87–3.18)	0.127	1.99 (1.26–3.15)	0.003
T3/4	4.05 (1.66–9.91)	0.002	3.57 (1.48–8.63)	0.005	3.67 (1.99–6.77)	< 0.001
**N stage**						
N0	3.49 (1.71–7.11)	< 0.001	2.22 (1.23–4.01)	0.008	2.70 (1.72–4.24)	< 0.001
N1	1.91 (0.85–4.29)	0.12	2.63 (0.93–7.45)	0.068	2.22 (1.21–4.09)	0.01
**M stage**						
M0	2.94 (1.49–5.81)	0.002	2.33 (1.26–4.31)	0.007	2.57 (1.64–4.04)	< 0.001
M1	4.34 (1.61–11.75)	0.004	3.11 (1.12–8.65)	0.03	3.87 (1.89–7.92)	< 0.001

### Building and Validating a Predictive Nomogram

**FIGURE 4:**
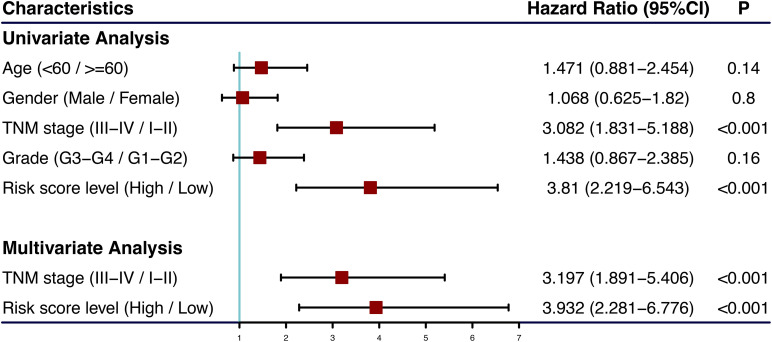
Forest plot of clinical characters and lncRNA signature in univariate and multivariate Cox analysis. The coordinate of red square represents the hazard ratio, and the length of the line represents the 95% confidence intervals.

**FIGURE 5:**
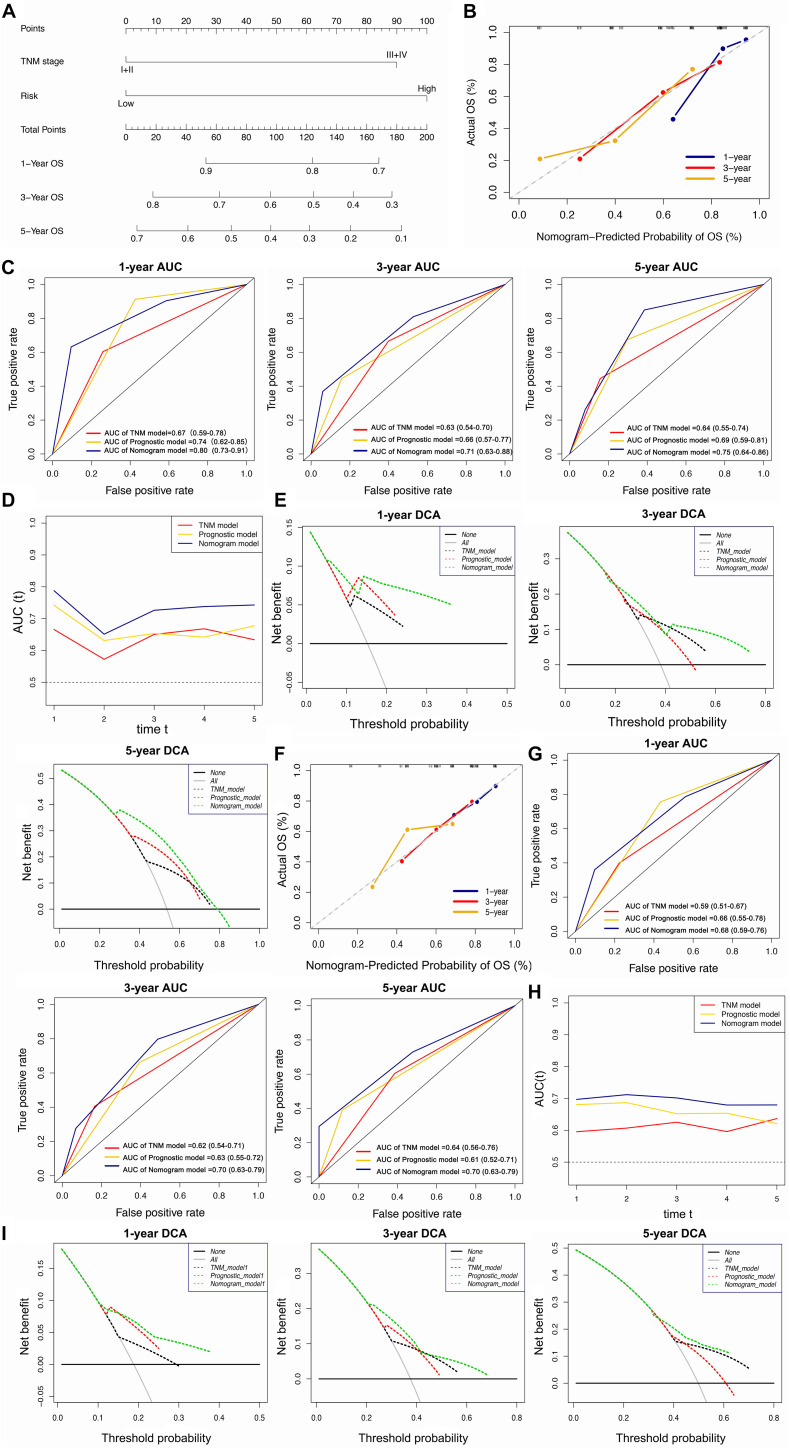
Construction and validation of the lncRNA-clinicopathologic nomogram for predicting the survival rate of HCC patient. **(A)** Development of a nomogram for predicting 1, 3, and 5 year survival rate of HCC patients. **(B)** Calibration curve of the nomogram. **(C)** ROC curve of TNM model, lncRNA signature, and nomogram for 1, 3, and 5 year OS of HCC patients. **(D)** The curves of time-dependent AUCs versus time (1–5 years) of TNM model, lncRNA signature, and nomogram. **(E)** DCA curves comparing the nomogram with other two models for 1, 3, and 5 year OS of HCC patients. **(A–E)** Data were from the training cohort. **(F)** Calibration curve of the nomogram. ROC curve of TNM model, lncRNA signature, and nomogram for 1, 3, and 5 year OS of HCC patients. **(G)** ROC curve of TNM model, lncRNA signature, and nomogram for 1, 3, and 5 year OS of HCC patients. **(H)** The curves of time-dependent AUCs versus time (1–5 years) of TNM model, lncRNA signature, and nomogram. **(I)** DCA curves comparing the nomogram with other two models for 1, 3, and 5 year OS of HCC patients. **(F–I)** Date were from the validation cohort.

The univariate and multivariate Cox regression analyses showed that TNM stage and our prognostic signature were both independent prognostic factors of HCC (Figure 4); therefore, these two factors were combined to construct subsequent nomograms (Figure 5A). The calibration curve of the nomogram demonstrated the best performance for predicting 3 year OS (Figure 5B). The C-indexes were 0.60, 0.65, and 0.69 for the TNM, the prognostic and the nomogram models, respectively. The AUCs of the nomogram for predicting 1-, 3-, and 5 year OS were 0.801, 0.714, and 0.749, respectively (Figure 5C), which were higher than those of the other models over time, suggesting that combining the lncRNA signature with the TNM stage increased the accuracy in predicting prognostic outcomes (Figure 5D). In addition, the DCA demonstrated that this nomogram had good clinical utility, which meant that using the nomogram to predict prognosis at 1, 3, or 5 years exceeded the performance of the independent predictors using the prognostic signature or the TNM stage (Figure 5E).

The calibration curve of the nomogram for predicting 1- and 3 year OS in the validation cohort, showed great consistency between prediction and actual observation (Figure 5F). The C-indexes were 0.57, 0.63, and 0.63 for the TNM, the prognostic and the nomogram models, respectively. The AUCs of the nomogram for predicting 1-, 3-, and 5 year OS were 0.676, 0.695, and 0.696, respectively (Figure 5G), which were higher than those of the other models over time (Figure 5H). The DCA suggested that using this nomogram brought more net benefit (Figure 5I).

In summary, the nomogram consisting of our lncRNA signature and a TNM model presented high accuracy in predicting the 1-, 3-, and 5 year OS of HCC patients in both the training and validation cohort. Using this nomogram to predict both short- and long-term survival rates for HCC patients might bring greater clinical benefit.

### PPI Network Analysis and Survival Analysis of Hub Genes

**FIGURE 6:**
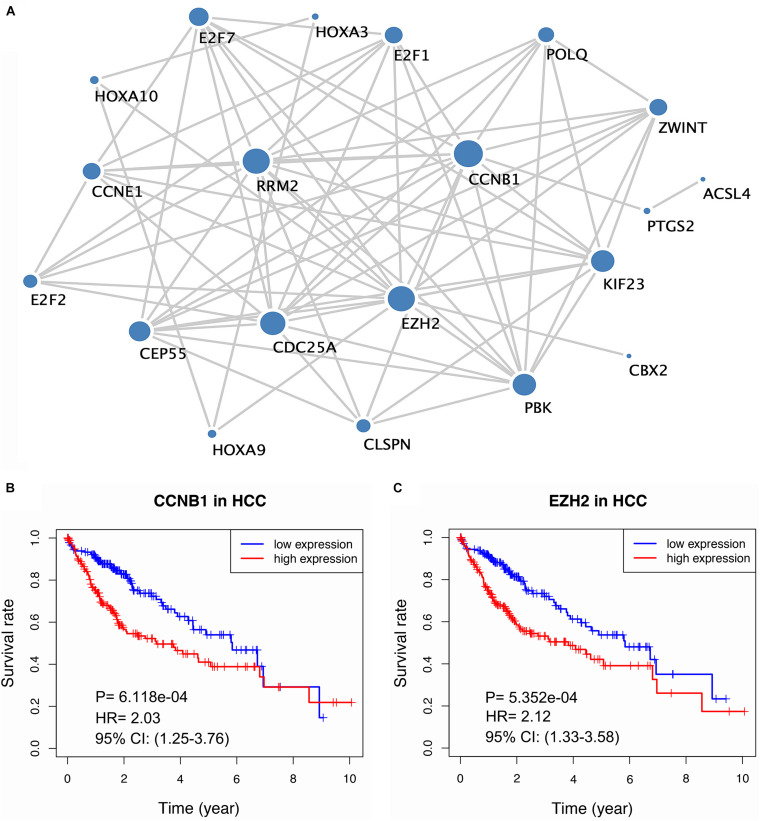
Build of the PPI network and survival analysis of the hub gene. **(A)** The PPI network for mRNA mediated by lncRNA signature. **(B–C)** Kaplan-Meier curve of two hub genes (CCNB1, EZH2).

To better comprehend the biological function of the targeted mRNAs mediated by the lncRNA signature, we established a PPI network by STRING (Figure 6A and Supplementary Table 11). After conducting topological feature analysis for the PPI network, *CCNB1* and *EZH2* were determined to be ranked in the top 3 (Supplementary Table 12), suggesting that they may be hub genes. Subsequently, we explored the association between the expression of hub genes and HCC prognosis. The results showed that high expression of *CCNB1* or *EZH2* is related to poor prognosis (Figure 6B–C), indicating that these two hub genes may have oncogenic effects.

### Validation of the lncRNA Signature and Hub Gene

**FIGURE 7:**
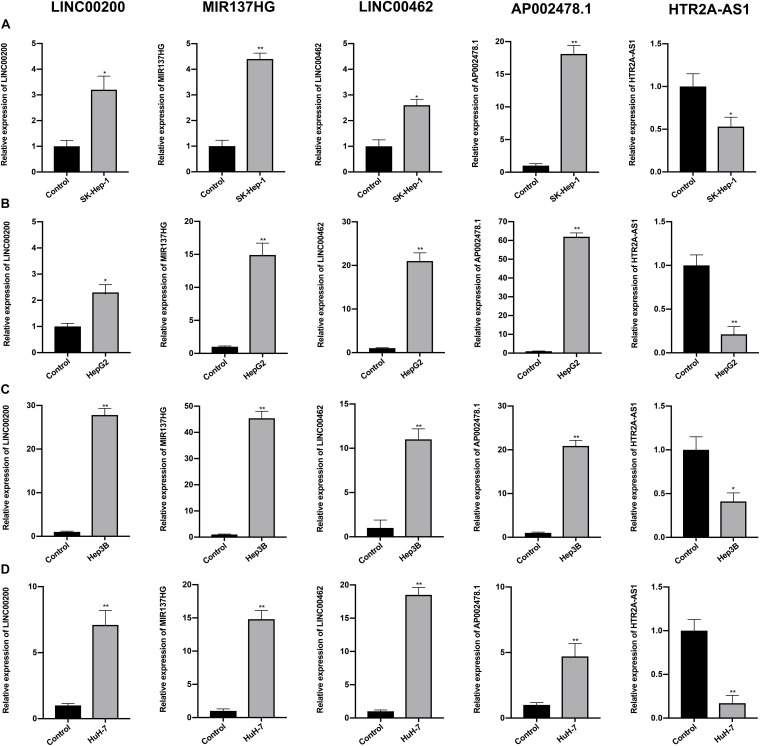
Expression of the five prognostic lncRNAs was detected by qRT-PCR. **(A)** Expression of five lncRNAs were detected in SK-Hep-1 cell lines. **(B)** Expression of five lncRNAs were detected in HepG2 cell lines. **(C)** Expression of five lncRNAs were detected in Hep3B cell lines. **(D)** Expression of five lncRNAs were detected in HuH-7 cell lines. Data are presented as mean ± SD, *P < 0.05 and **P < 0.01.

**FIGURE 8:**
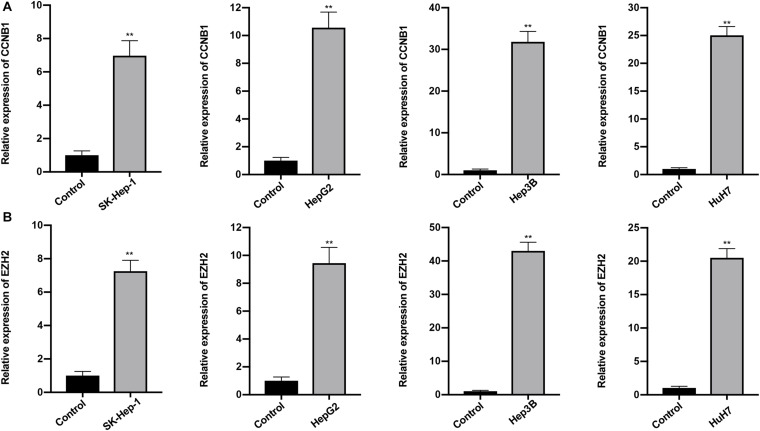
Expression of CCNB1 and EZH2 was detected by qRT-PCR. **(A)** Expression of CCNB1 in four HCC cell lines. **(B)** Expression of EZH2 in four HCC cell lines. Data are presented as mean ± SD, ^∗∗^*P* < 0.01.

The expression profiles of the five lncRNAs comprising the prognostic signature were verified in HCC and liver cell lines by qRT-PCR. HTR2A-AS1 was downregulated in 4 HCC cell lines, while LINC00200, MIR137HG, LINC00462, and AP002478.1 were upregulated in 4 HCC cell lines as shown in Figures 7A–D. These results demonstrated that these lncRNAs might be involved in the development of HCC. In particular, HTR2A-AS1 may play a protective role in HCC development. Additionally, the expression of hub genes (*CCNB1* and *EZH2*) was detected (Figures 8A,B). These results showed that these two genes were both upregulated in HCC cell lines, which was consistent with our survival analysis, concurrently suggesting a tight association between the dysregulation of these two genes and HCC development.

## Discussion

Hepatocellular carcinoma (HCC), the most common form of primary liver cancer, typically develops on the background of chronic liver disease and is an aggressive disease with a poor prognosis (Craig et al., 2019). A large number of lncRNAs have been associated with HCC with the development of genome-wide sequencing technologies in recent years. Recent studies have demonstrated that the dysregulation of lncRNAs could regulate gene expression by competitively binding miRNAs, subsequently affecting cell proliferation, apoptosis and metastasis, and eventually leading to cancer development. Thus finding lncRNA molecular markers for prognosis and treatment is necessary considering their vital roles in HCC.

In the present study, clinical information and RNA-seq data for HCC were obtained from the TCGA database. Differential expression analysis was then conducted comparing normal liver tissues and HCC tissues to identify mRNAs, lncRNAs, and miRNAs. The combination of these coding and non-coding genes was used for the re-construction of the ceRNA network. In addition, functional enrichment analysis was performed to explore the biological function of lncRNAs included in the ceRNA network. The results of GO enrichment analysis showed that these lncRNAs can mediate mitotic cell cycle-related pathways to promote oncogenic cell proliferation and tumor growth (Schmitt and Chang, 2016). Additionally, KEGG analysis revealed that these lncRNAs can intervene in several classic tumor-related signaling pathways, such as pathways in cancer, cell cycle, and the p53 signaling pathway. Besides, our results also suggested that these lncRNAs may participate in the hepatitis B-associated pathway to affect HCC progression. Furthermore, five survival-related lncRNAs were identified using univariate and multivariate Cox regression analysis, and these molecules were subsequently selected for the development of a prognostic signature to accurately predict the prognosis of the HCC patients. The AUC value of the ROC curve for 1-, 3-, and 5 year OS suggested that our lncRNA signature has high predictive power for both short- and long-term survival. Notably, a previous study reported a 13-lncRNA signature that had a good ability for HCC prognosis prediction (Bai et al., 2019). Interestingly, four out of the above lncRNAs (MIR137HG, LINC00462, HTR2A-AS1, and AP002478.1) were common with ours. The result of current study suggested that our lncRNA signature could still maintain a good predictive power for HCC prognosis by curtailing the lncRNA numbers, which indicated that this five-lncRNA signature may be more convenient and flexible. Some of our lncRNA biomarkers have been reported to play critical roles in cancer biology. For instance, one study demonstrated that LINC00462 was upregulated in HCC tissues and that knocking down the LINC00462 in HCC cells could result in a considerably less aggressive oncogenic phenotype (Gong et al., 2017). Zhou et al. revealed that the aberrant expression of LINC00462 is linked with pancreatic cancer, and high levels of LINC00462 were associated with poor prognosis of pancreatic cancer. This study demonstrated that the upregulation of LINC00462 could boost cell proliferation by promoting the cell cycle process while inhibiting cell apoptosis and adhesion *in vitro*. In addition, LINC00462 can promote the cancer cell migration and the invasion by promoting the epithelial-mesenchymal transition (Zhou et al., 2018). Guan et al. (2020) suggested that HTR2A-AS1 is downregulated in HCC cells, and upregulating the expression of HTR2A-AS1 can induce apoptosis and inhibit the proliferation, invasion and migration of HCC cells. In particular, according to the aforementioned reports, LINC00462 and HTR2A-AS1 may possess oncogenic and carcinostatic roles, respectively, which is consistent with our results that LINC00462 is related to poor prognosis, while HTR2A-AS1 is associated with favorable prognosis. However, limited evidence exists for the detailed biological function of other lncRNAs in our prognostic signature. Hence, the potential mechanism of these lncRNAs has not been elucidated and further research with functional experiments is required.

Traditionally, as a systematic anatomic-based classification, the TNM stage provides a method to estimate cancer prognosis (Huang and O’Sullivan, 2017). Recently, with the development of precision medicine, accumulating evidence demonstrated that lncRNAs possess a potential predictive value in cancer prognosis (Angenard et al., 2019; Li et al., 2019; Xu et al., 2019). Thus, it was shown that the prognostic model generated by combining lncRNAs with TNM stage provides higher predictive ability compared to currently available prognostic systems. Here, we constructed a lncRNA-clinicopathological nomogram consisting of TNM stage and lncRNA signature. The calibration curve showed that the predicted OS by the nomogram had good consistency with the actual OS, especially in the 3 year OS, which indicated that this nomogram had excellent prediction performance. In addition, the C-index and AUC of the ROC curve showed that the nomogram performed better in prognosis in comparison to the individual prognostic factors, such as the TNM stage. This result suggested that combining the lncRNA signature with clinicopathological factors can promote the prediction power. Additionally, the DCA demonstrated that using the nomogram to predict patient prognosis, especially in long-term survival, can bring more net benefit. Thus, the nomogram might have good prospects in clinical applications.

Hub genes are often responsible for the regulation of other genes in related pathways and they play a crucial role in biological processes. Thus, we constructed a PPI network to better understand the mRNAs involved in the ceRNA network and affected by the lncRNA signature. Two hub genes (*CCNB1* and *EZH2*) were identified after calculating the topological feature parameter of genes involved in the PPI network (Scardoni et al., 2009). These two genes have been well studied. *CCNB1* is one of the cyclin family members that regulates the G2/M transition of the cell. Several studies have proven the prognostic value of *CCNB1*. For instance, high expression of *CCNB1* was associated with poor prognosis in breast and prostate cancers (Aaltonen et al., 2009; Ersvaer et al., 2019). Interestingly, several studies revealed the opposite role of *CCNB1*; colorectal cancer tissue has a higher expression level of *CCNB1* than normal samples, and this elevated expression level was positively associated with survival, suggesting the heterogeneity of *CCNB1* in different tumor biological processes. In addition, *in vitro* experiments showed that high levels of *CCNB2* can suppress the expression of E-cadherin to inhibit the invasion and metastasis of tumor cells (Fang et al., 2015; Chen et al., 2019). Another key gene, *EZH2*, is a histone methyltransferase subunit of a polycomb repressive complex, which is highly expressed in numerous cancers (Kim and Roberts, 2016). Prior evidence has demonstrated that the overexpression of *EZH2* is a marker of advanced and metastatic disease in many solid tumor (Chase and Cross, 2011). Additionally, immunohistochemical examination has indicated that *EZH2* may be a promising biomarker for the diagnosis of HCC (Cai et al., 2011). Importantly, we verified that *CCNB1* and *EZH2* were overexpressed in HCC cells by conducting qRT-PCR. On parallel, survival analysis showed that these two genes were associated with poor prognosis. Our results are consistent with the findings of previous reports. Although *CCNB1* and *EZH2* were largely not reported in HCC, our findings suggest that they may play crucial roles in the progression of HCC. Thus, further research to investigate the biological function of these two genes is needed.

Although the lncRNA signature performs well in HCC prognosis, several limitations should be noted. This study has the advantage that the lncRNA prognostic model was constructed and validated by a massive cohort from the TCGA database, but it still employs a retrospective design. Hence, the validation of this lncRNA signature in the large prospective cohort is required. Additionally, further functional studies are required as the biological function of the lncRNA signature and two hub genes have not been fully characterized.

## Conclusion

A more accurate ceRNA network has been reconstructed by employing rigorous screening processes and it could provide a new insight into the identification of potential regulation mechanisms for the development of HCC. A five-lncRNA prognostic signature was reconstructed based on this network, demonstrating high performance in predicting the survival of HCC patients. In addition, two hub genes, *CCNB1* and *EZH2*, have been identified after performing PPI analysis for lncRNA signature-mediated mRNA, which may have crucial effects on HCC progression. The five lncRNAs (LINC00200, MIR137HG, LINC00462, HTR2A-AS1, and AP002478.1) and two hub genes (*CCNB1* and *EZH2*) may be potential therapeutic targets. More importantly, we have introduced a well-executed nomogram that exhibited strong potential for clinical applications.

## Data Availability Statement

Publicly available datasets were analyzed in this study. This data can be found here: https://portal.gdc.cancer.gov/.

## Ethics Statement

As our data were downloaded from the TCGA database, there is no requirement for ethics committee approval and consent to participate.

## Author Contributions

J-HS designed the study and revised the manuscript. J-RS, C-FK, K-MX, X-KQ, and J-LY collected and assembled the data. J-RS, C-FK, and K-MX conducted the experiment. J-RS and C-FK performed data analysis and interpretation. J-RS drafted the manuscript. All the authors read and approved the final manuscript.

## Conflict of Interest

The authors declare that the research was conducted in the absence of any commercial or financial relationships that could be construed as a potential conflict of interest.

## Footnotes

1 https://portal.gdc.cancer.gov, version 17.0.

2 https://david.ncifcrf.gov/, version 6.8.

3 https://string-db.org, version 11.0.

## Supplementary Material

Supplementary Figure 1 Volcano plot and heatmap of differentially expressed lncRNAs, miRNAs, and mRNAs for HCC. **(A)** Volcano plot of differentially expressed genes. **(B)** Heatmap of differentially expressed genes.

Supplementary Figure 2 LncRNA-miRNA-mRNA ceRNA network for HCC. The red diamond, square, and round represent up-regulated lncRNA, miRNA, and mRNA, respectively. The blue diamond, square, and round represent down-regulated lncRNA, miRNA, and mRNA, respectively.

Supplementary Table 1 The primers sequences of lncRNAs and mRNAs.

Supplementary Table 2 Clinical characteristics of 376 patients with hepatocellular carcinoma (HCC).

Supplementary Table 3 DElncRNAs.

Supplementary Table 4 DEmiRNAs.

Supplementary Table 5 DEmRNAs.

Supplementary Table 6 The lncRNA-miRNA interaction pairs predicted by miRcode.

Supplementary Table 7 The miRNA-mRNA interaction pairs predicted by MiRDB, miRTarBase, and Targetscan.

Supplementary Table 8 Univariate Cox analysis of lncRNAs in the ceRNA network.

Supplementary Table 9 Risk score of train and test groups.

Supplementary Table 10 1-, 3-, and 5 year OS baseline information.

Supplementary Table 11 Topological details for the PPI network.

Supplementary Table 12 Topological features for each gene in PPI network.

## Abbreviations

HCC: hepatocellular carcinoma
ceRNA: competing endogenous RNA
lncRNA: long non-coding RNA
miRNA: microRNA
TCGA: The Cancer Genome Atlas
TMM: trimmed mean of *M*-values
AUC: The area under curve
ROC: receiver operating characteristic
DAVID: The Database for Annotation, Visualization and Integrated Discovery
GO: Gene Ontology
KEGG: Kyoto Genome Encyclopedia
PPI: protein-protein interaction
OS: overall survival
qRT-PCR: quantitative real-time PCR
