# Supplementary figures and images for: Integrated Analysis of lncRNA-Mediated ceRNA Network Reveals a Prognostic Signature for Hepatocellular Carcinoma
Source: Front Genet. 2020 Dec 14;11:602542. doi: 10.3389/fgene.2020.602542 (PMC7767998; doi:10.3389/fgene.2020.602542)

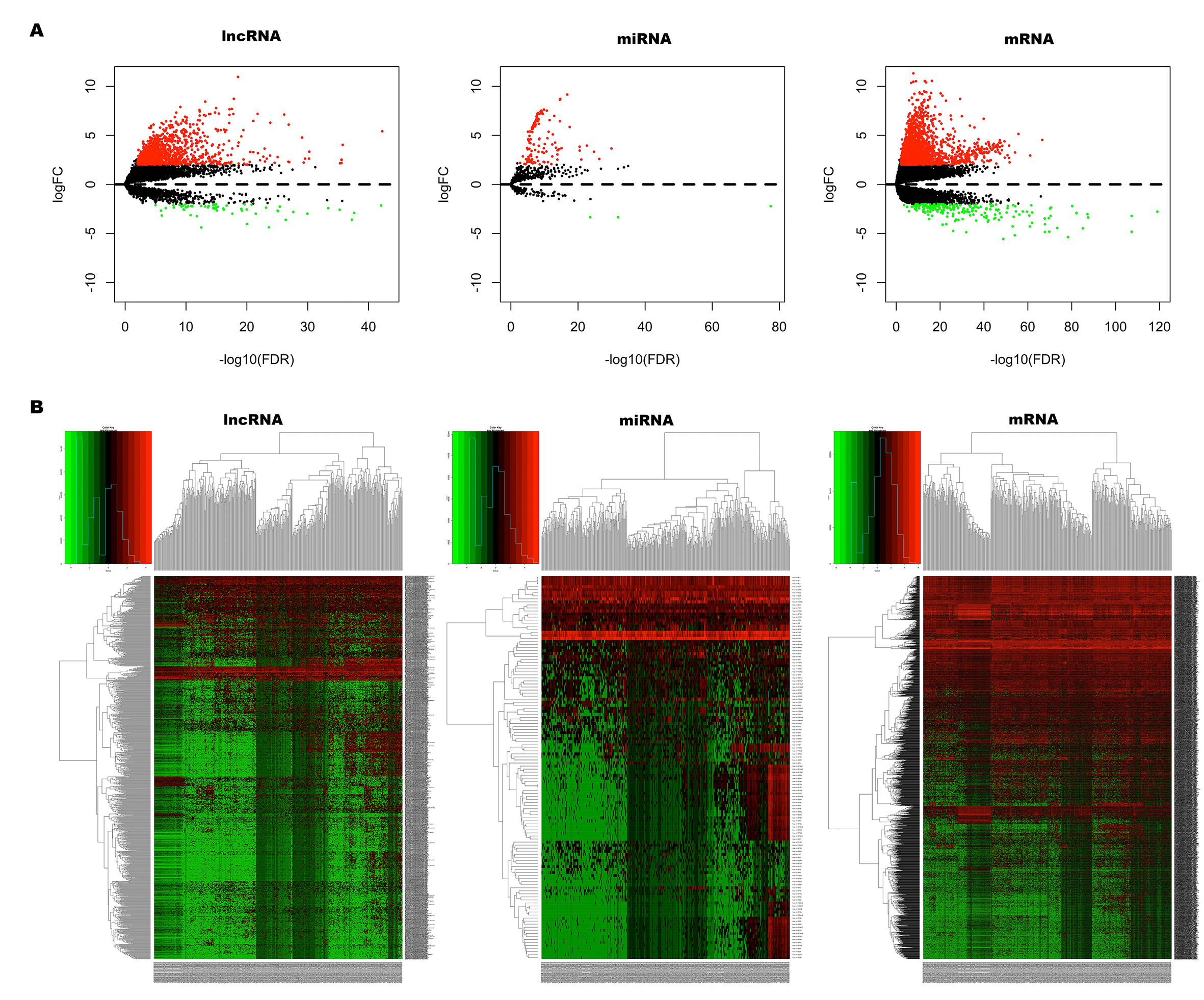

Supplement: Supplementary Figure 1 — Volcano plot and heatmap of differentially expressed lncRNAs, miRNAs, and mRNAs for HCC. (A) Volcano plot of differentially expressed genes. (B) Heatmap of differentially expressed genes. [file Image_1.TIF]

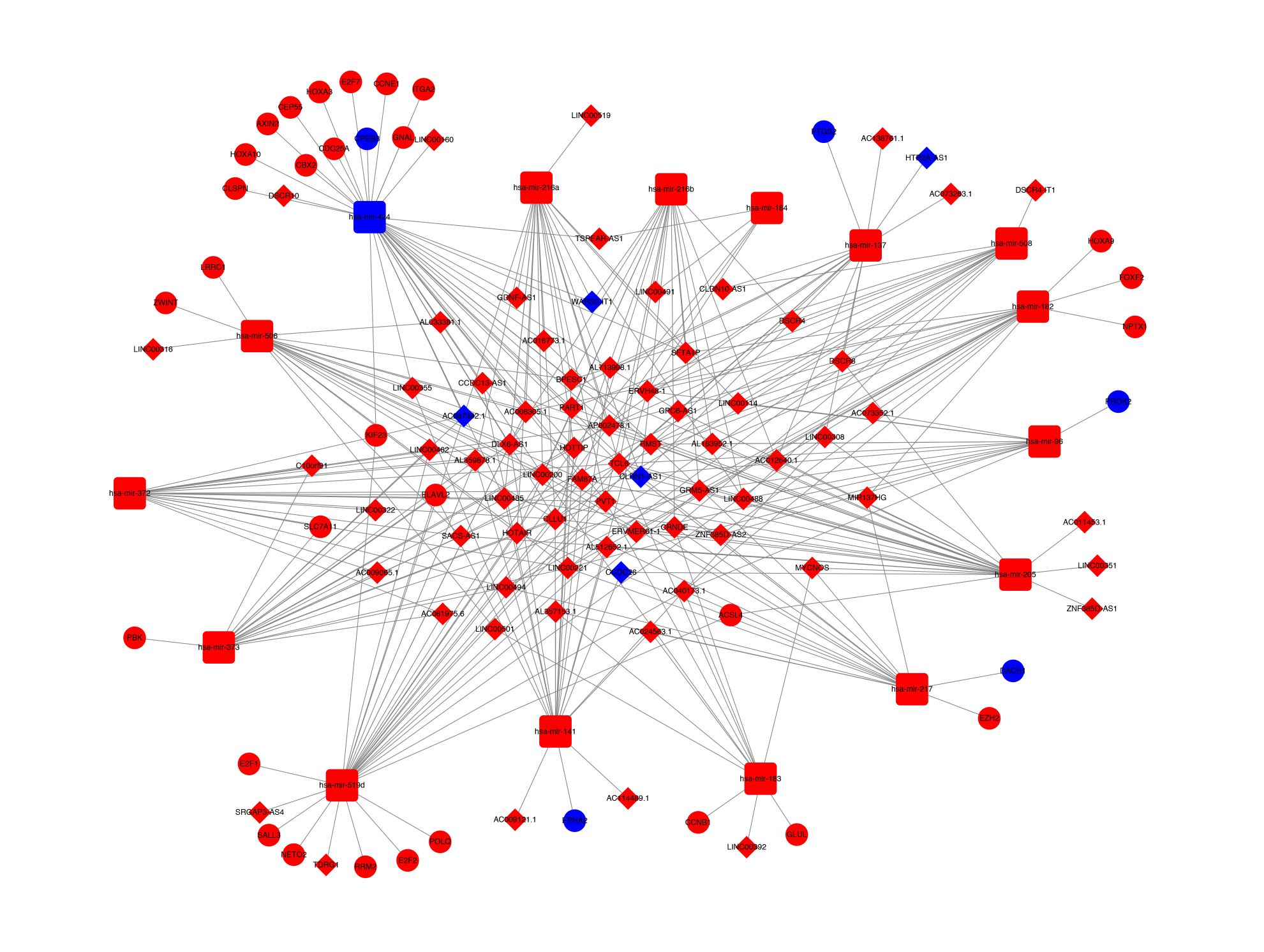

Supplement: Supplementary Figure 2 — LncRNA-miRNA-mRNA ceRNA network for HCC. The red diamond, square, and round represent up-regulated lncRNA, miRNA, and mRNA, respectively. The blue diamond, square, and round represent down-regulated lncRNA, miRNA, and mRNA, respectively. [file Image_2.TIF]
